# Roles of Mitogen-Activated Protein Kinases in Osteoclast Biology

**DOI:** 10.3390/ijms19103004

**Published:** 2018-10-01

**Authors:** Kyunghee Lee, Incheol Seo, Mun Hwan Choi, Daewon Jeong

**Affiliations:** Department of Microbiology, Laboratory of Bone Metabolism and Control, Yeungnam University College of Medicine, Daegu 42415, Korea; kyungheelee@ynu.ac.kr (K.L.); htr@daum.net (I.S.); choibak@ynu.ac.kr (M.H.C.)

**Keywords:** mitogen-activated protein kinases (MAPKs), MAPK kinetics, osteoclast differentiation, bone remodeling

## Abstract

Bone undergoes continuous remodeling, which is homeostatically regulated by concerted communication between bone-forming osteoblasts and bone-degrading osteoclasts. Multinucleated giant osteoclasts are the only specialized cells that degrade or resorb the organic and inorganic bone components. They secrete proteases (e.g., cathepsin K) that degrade the organic collagenous matrix and establish localized acidosis at the bone-resorbing site through proton-pumping to facilitate the dissolution of inorganic mineral. Osteoporosis, the most common bone disease, is caused by excessive bone resorption, highlighting the crucial role of osteoclasts in intact bone remodeling. Signaling mediated by mitogen-activated protein kinases (MAPKs), including extracellular signal-regulated kinase (ERK), c-Jun N-terminal kinase (JNK), and p38, has been recognized to be critical for normal osteoclast differentiation and activation. Various exogenous (e.g., toll-like receptor agonists) and endogenous (e.g., growth factors and inflammatory cytokines) stimuli contribute to determining whether MAPKs positively or negatively regulate osteoclast adhesion, migration, fusion and survival, and osteoclastic bone resorption. In this review, we delineate the unique roles of MAPKs in osteoclast metabolism and provide an overview of the upstream regulators that activate or inhibit MAPKs and their downstream targets. Furthermore, we discuss the current knowledge about the differential kinetics of ERK, JNK, and p38, and the crosstalk between MAPKs in osteoclast metabolism.

## 1. Introduction

Normal bone physiology depends on the coupled processes of removing old bone and replacing it with new bone [1]. Throughout life, bone undergoes continuous remodeling through concerted bone matrix formation and mineralization (anabolic process) by osteoblasts and mineralized bone matrix degradation (catabolic process) by osteoclasts [2]. Osteoclasts are multinucleated cells that are formed from monocyte/macrophage lineage cells, and their differentiation and function are regulated by various cytokines, hormones, and growth factors [3,4]. Especially, macrophage colony stimulating factor (M-CSF) and receptor activator for nuclear factor κ-B ligand (RANKL) are indispensable in the regulation of the sequential processes of osteoclastogenesis, including osteoclast precursor proliferation, adhesion, migration, and cell-cell fusion to form multinucleated cells, as well as in the migration, survival, and bone-resorptive function of mature osteoclasts [5]. Both M-CSF and RANKL act through mitogen-activated protein kinases (MAPKs), extracellular signal-regulated kinase (ERK), c-Jun N-terminal kinase (JNK), and p38 signaling during osteoclast differentiation and bone resorption. MAPK signaling activated by M-CSF is mainly involved in the regulation of osteoclast precursor proliferation, whereas RANKL-induced MAPK activation is primarily implicated in osteoclast differentiation [6,7]. Moreover, a recent, advanced study revealed that the activation of MAPKs by M-CSF or RANKL differs in terms of the extent, duration, and isoform specificity of MAPK phosphorylation, thus determining the distinct cell fates of proliferation or differentiation in osteoclast precursors [8]. In osteoclast precursors, two ERK forms, ERK1/2, and three JNK isoforms, JNK1/2/3, are mainly involved in osteoclast precursor proliferation and osteoclast apoptosis, respectively [4,8,9]. Among the four isoforms of p38 (α, β, γ, and δ), p38α is highly expressed in osteoclast precursors and mature osteoclasts and plays a key role in osteoclast differentiation and bone resorption [10].

MAPKs convert a variety of extracellular stimuli into specific cellular responses, thus acting as signaling hubs, in eukaryotic cells [11]. MAPK pathways are organized into three-tiered cascades comprised of three molecules: MAPK, MAPK kinase (MAPKK or MEK), and MAPKK kinase (MAPKKK or MEKK). In the phosphorelay system, MAPKKKs, which are serine/threonine protein kinases, phosphorylate and activate MAPKKs, which then dually phosphorylate the threonine and tyrosine residues of the conserved TXY motif (“X” stands for glutamic acid, proline, or glycine) of the activation loop of MAPKs, including ERK, JNK, and p38 [12]. The activities of MAPK pathways are properly regulated through dephosphorylation of the threonine or tyrosine residue of the TXY motif by phosphatases, such as dual-specificity phosphatases (DUSPs), which counter the activities of kinases [13]. The activation of MAPK pathways leads to various biological outcomes, including gene induction, cell proliferation and survival, apoptosis, and differentiation, as well as cellular stress and inflammatory responses.

The signaling transmission of extracellular stimuli via MAPK activation to appropriate intracellular molecules has been established to be essential for the regulation of osteoclast differentiation and bone remodeling. Numerous studies exploring the roles of MAPKs in osteoclast metabolism have suggested that ERK, JNK, and p38 are key players in osteoclast differentiation and activation. In this review, we describe the peculiar roles of MAPKs in osteoclast metabolism, as well as various upstream stimulators and inhibitors of MAPKs and their downstream targets. In addition, we discuss current knowledge regarding the distinctive kinetics of MAPKs and the crosstalk between MAPKs in osteoclast metabolism.

## 2. ERK Signaling in Osteoclasts

**Figure 1:**
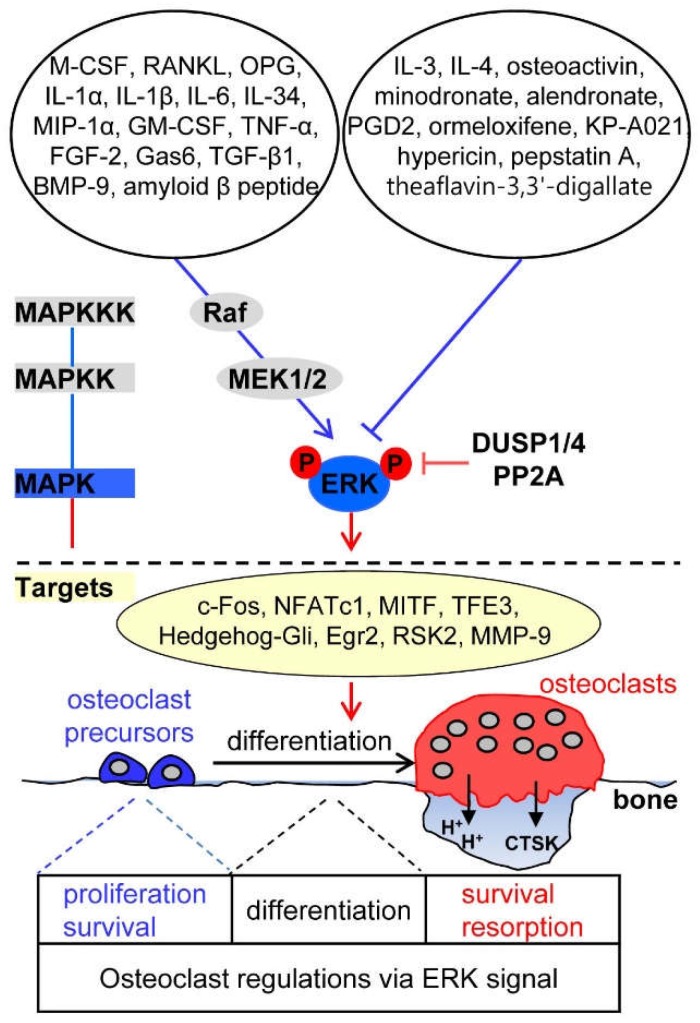
Osteoclastogenic signaling cascade controlled by upstream activators and inhibitors of extracellular signal-regulated kinase (ERK). The marked molecules are described in the text entitled “ERK signaling in osteoclasts”. CTSK, cathepsin K; M-CSF, macrophage colony-stimulating factor; RANKL, receptor activator of nuclear factor κ-B ligand; OPG, osteoprotegerin; IL, interleukin; MIP, macrophage inflammatory proteins; GM-CSF, granulocyte-macrophage colony-stimulating factor; TNF, tumor necrosis factor; FGF, fibroblast growth factor; TGF, transforming growth factor; BMP, bone morphogenetic protein; PGD2, prostaglandin D2; MAPK, mitogen-activated protein kinase; MAPKK, mitogen-activated protein kinase kinase; MAPKKK, mitogen-activated protein kinase kinase kinase; DUSP, dual-specificity phosphatase. Arrows indicate activation of the signaling pathways while T bars indicate inhibition of the signaling pathways.

The ERK signaling pathway has been implicated in the survival, proliferation, apoptosis, formation, polarity, podosome disassembly, and differentiation of osteoclasts. Combined findings in ERK1 knockout and hematopoietic ERK2 conditional knockout mice showed that ERK1 plays a crucial role in modulating osteoclast differentiation, migration, and bone resorption [14]. A variety of cytokines, growth factors, and hormones positively or negatively regulate ERK signaling in osteoclasts (Figure 1). The ERK signaling cascade consists of a core of three serially phosphorylating protein kinases. The activation of Raf isoforms via Ras-Raf interaction stimulates the MAPKKs MEK1 and MEK2, which then activate ERK1 and ERK2 by dual phosphorylation at the conserved Thr-Glu-Tyr (TEY) motif [15,16], which leads to the phosphorylation of various downstream substrates, including transcription factors.

Recently, the functions of less-well-studied MEK5/ERK5 signaling pathways in bone biology begin to be of interest [17,18]. It was reported that conditional deletion of ERK5 in the mouse prostate using *Nkx3.1-Cre* recombinase expression resulted in a severely deformed and curved spine, with an associated loss of trabecular bone volume [17]. These spinal abnormalities in *Nkx3.1-Cre* ERK5 null mice are associated with increased osteoclast activity. In addition, M-CSF, but not RANKL, induces ERK5 phosphorylation and the consequent M-CSF/MEK5/ERK5 signaling mediates osteoclast differentiation [19].

### 2.1. Upstream Activators of ERK Signaling in Osteoclasts

The osteoclastogenic factors M-CSF and RANKL play a critical role in osteoclast differentiation by inducing the phosphorylation of ERK1 and ERK2 [4]. The binding of M-CSF to its receptor c-Fms results in the phosphorylation of specific tyrosine residues of c-Fms. The phosphorylated site at the intracellular cytosolic tail of c-Fms interacts with growth factor receptor-binding protein-2, a stimulator of the Ras/Raf pathway, which then leads to the activation of ERK1 and ERK2, enhancing osteoclast precursor proliferation and survival [20,21]. Binding of RANKL to its receptor RANK leads to the recruitment of the adaptor protein, TNF receptor-associated factor 6 (TRAF6), to the cytoplasmic tail in a submembrane compartment and then triggers ERK activation. RANKL/RANK/TRAF6/ERK cascades have been shown to regulate osteoclast formation and function [22,23]. Interestingly, osteoprotegerin (OPG), a decoy receptor that binds to RANKL, and thus, suppresses osteoclast differentiation by interrupting the interaction between RANKL and RANK, can also phosphorylate ERK1 and ERK2 and directly induce podosome disassembly in osteoclasts [22,24,25].

Several reports have suggested that ERK activation by inflammatory cytokines positively regulates osteoclastogenesis. Interleukin-1β (IL-1β) acts synergistically with RANKL to increase ERK activation in a Ca^2+^-dependent manner [26] and IL-1α, IL-6, and IL-34 induce phosphorylation of ERK1 and ERK2, leading to the promotion of osteoclastogenesis [27,28,29]. Macrophage inflammatory protein-1α (MIP-1α) secreted from multiple myeloma cells induces osteoclast formation by activating the MEK/ERK/c-Fos pathway [30]. Granulocyte-macrophage colony-stimulating factor (GM-CSF)-induced ERK activation promotes the fusion of mononuclear osteoclasts to form multinucleated osteoclasts by inducing the expression of dendritic cell-specific transmembrane protein (DC-STAMP, also known as TM7SF4) via the Ras/ERK pathway [31].

Growth factors, such as fibroblast growth factor-2 (FGF-2), growth arrest-specific gene 6 (Gas6), and tumor necrosis factor-α (TNF-α), stimulate mature osteoclast function and survival through ERK activation [32,33]. ERK is transiently activated during transforming growth factor-β1 (TGF-β1)-induced apoptosis of osteoclasts differentiated from human umbilical cord blood monocytes, via the activation of caspase-9 and upregulation of the pro-apoptotic protein Bim [34]. The binding of bone morphogenetic protein-9 (BMP-9) to its receptor anaplastic lymphoma kinase 1 on the cell surface activates the canonical Smad-1/5/8 pathway and the ERK pathway, and supports the formation, function, and survival of osteoclasts derived from human umbilical cord blood monocytes [35]. Interestingly, in patients with Alzheimer’s disease, who have a high risk of osteoporotic hip fracture, amyloid beta peptide, one of the pathological hallmarks of Alzheimer’s disease that is abnormally deposited in bone tissues [36], was shown to enhance RANKL-induced ERK and NF-κB activation and to promote osteoclastic bone resorption [37]. Taken together, various upstream stimulators of ERK pathway were found to positively regulate the process of osteoclast differentiation.

### 2.2. Upstream Inhibitors of ERK Signaling in Osteoclasts

IL-3 and IL-4, known as anti-osteoclastogenic cytokines, suppress osteoclastogenesis and/or osteoclastic bone resorption via inhibition of the ERK pathway and activation of signal transducer and activator of transcription 5 (STAT5) [38,39,40]. Prostaglandin D2 inactivates ERK signaling during chemoattractant receptor homologous molecule expressed on T-helper type 2 cells (CRTH2)-mediated apoptosis of osteoclasts derived from human peripheral blood mononuclear cells [41]. In osteoactivin-CD44-ERK signal cascades, shedding of the ectodomain of osteoactivin, a heavily glycosylated type I transmembrane protein that is expressed in both osteoclasts and osteoblasts, produces a soluble form of osteoactivin [42] that binds to the CD44 receptor, followed by the inhibition of ERK signaling, and thus, decreased osteoclast differentiation [43].

Several pharmacological compounds, including anti-osteoporotic agents, have been reported to inhibit osteoclastogenesis by suppressing ERK signaling. Nitrogen-containing bisphosphonates, such as minodronate and alendronate, which are used as anti-resorptive drugs for the treatment of metabolic bone diseases [44], have been shown to decrease the phosphorylation of ERK1/2 and Akt, thereby inhibiting osteoclast formation [45]. Ormeloxifene, which is a nonsteroidal selective estrogen receptor modulator that exerts an estrogen-agonistic effect and has anticancer activity in breast cancer [46], has an anti-osteoclastogenic effect, resulting from ERK1/ERK2 and JNK inactivation by inhibiting the generation of RANKL-induced reactive oxygen species (ROS) [47]. KP-A021, a triazole-based compound that exhibits anti-inflammatory, anti-tumor, anti-tubercular, and anti-fungal activities, reportedly inhibits osteoclast differentiation by suppressing RANKL-induced MEK-ERK phosphorylation cascades [48,49]. Hypericin, a naphtodianthrone isolated from *Hypericum perforatum* and a potent and selective inhibitor of protein kinase C that reduces neuropathic pain, attenuates RANKL-induced osteoclastogenesis of bone marrow-derived macrophages via specific inhibition of the ERK signaling pathway without affecting JNK, p38, and NF-κB signaling in vitro, and suppresses titanium particle-induced bone erosion in vivo [50]. Pepstatin A, an inhibitor of aspartic proteinases, such as cathepsins D and E, and theaflavin-3,3′-digallate, a natural active compound derived from black tea, inhibit osteoclast formation and polarization and bone resorption by specifically suppressing RANKL-induced ERK signaling [51,52]. Therefore, inflammatory cytokines and pharmacological agents capable of inhibiting RANKL-induced ERK activation are regarded to negatively regulate osteoclast differentiation and function.

### 2.3. Downstream Targets of ERK Signaling in Osteoclasts

ERKs phosphorylate numerous downstream target substrates to control osteoclastogenesis. ERKs govern various transcription factors during osteoclastogenesis. c-Fos is phosphorylated at its C-terminal domain on serines 362 and 374 through ERK activation in response to M-CSF or RANKL [53,54]. The expression of c-Fos and nuclear factor of activated T-cells, cytoplasmic 1 (NFATc1), which are crucial osteoclastogenic transcription factors, is regulated via GM-CSF-induced ERK signaling [31]. M-CSF-stimulated ERK1 and ERK2 activation directly phosphorylates microphthalmia-associated transcription factor (MITF), a basic/helix-loop-helix/leucine-zipper transcription factor essential for osteoclast maturation, and its partner protein, transcription factor E3 (TFE3) [55]. Estrogen (17β-estradiol)-induced ERK activation inhibits osteoclastogenesis and promotes osteoclast apoptosis by downregulating signaling through the transcription factor Hedgehog-Gli. This fact implies that estrogen deficiency in postmenopausal osteoporosis induces increased and activated osteoclasts via the activation of Hedgehog-Gli signaling regulated by a MEK/ERK cascade [56]. M-CSF-induced, immediate gene induction of the Krüppel-like zinc finger transcription factor Egr2 in osteoclasts maintains osteoclast survival by inducing the pro-survival Bcl2 family member Mcl1 and proteolytic degradation of the pro-apoptotic Bim [57]. In addition, ribosomal S6 kinase 2 (RSK2), a member of the p90RSK family of serine/threonine kinases, is a downstream target of ERK1/ERK2 that participates in modulating M-CSF-induced PI3K/Akt activation through an ERK/RSK2-mediated negative feedback loop in macrophages [58]. RANKL-induced ERK activation induces the expression and activity of matrix metalloproteinase 9 (MMP-9, also termed gelatinase B/type IV collagenase), which is implicated in osteoclast migration and bone resorption [59], through TRAF6, but not TRAF2, in osteoclast precursors [60].

### 2.4. Phosphatase Regulation of ERK Signaling in Osteoclasts

DUSPs, protein phosphatases that dephosphorylate both phosphotyrosine and phosphoserine/phosphothreonine protein residues, play an important role in the duration, magnitude, and spatiotemporal regulation of MAPK activities [61]. STAT5, a member of the STAT family of transcription factors essential for cytokine-regulated processes [62], negatively regulates the activity of MAPKs, in particular, ERK1/2, by inducing the expression of *DUSP1* and *DUSP2*, thus suppressing the bone-resorbing activity of osteoclasts [39]. RANKL promotes osteoclastogenesis via sustained ERK activation, whereas RANKL together with the toll-like receptor 9 (TLR9) ligand, oligodeoxynucleotides containing unmethylated CpG dinucleotides (CpG-ODN), induces transient ERK activation by enhanced ERK dephosphorylation, due to the expression of phosphatase PP2A, a serine/threonine phosphatase, thus accelerating the degradation of osteoclastogenic transcription factor c-Fos and thereby inhibiting osteoclastogenesis [53].

## 3. JNK Signaling in Osteoclasts

**Figure 2:**
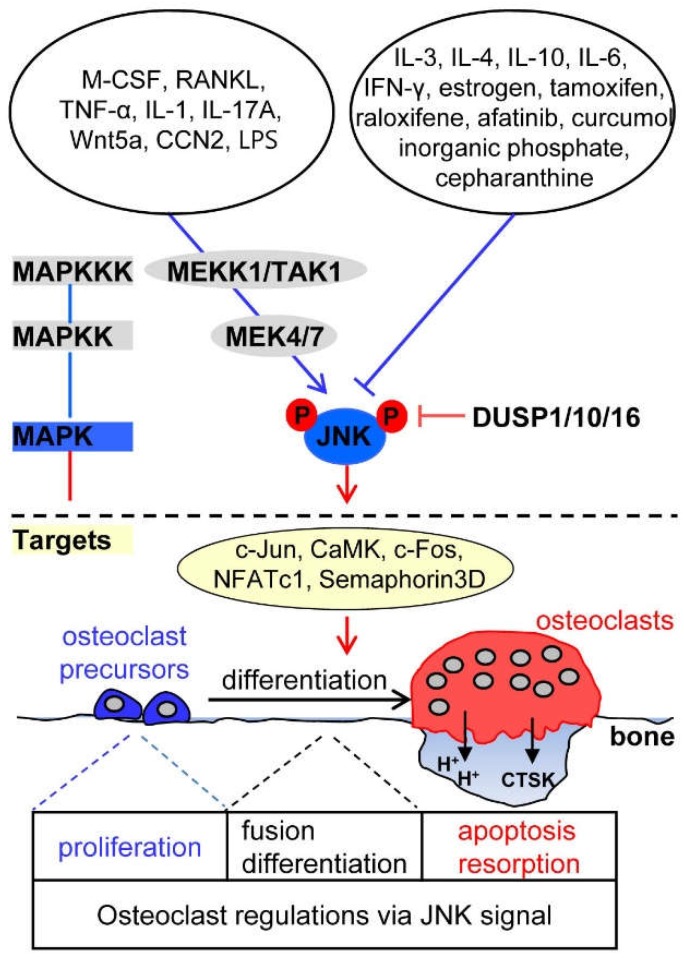
Osteoclastogenic signaling cascade controlled by upstream activators and inhibitors of c-Jun N-terminal kinase (JNK). The indicated molecules are described in the section of “JNK signaling in osteoclasts”. Arrows indicate activation of the signaling pathways while T bars indicate inhibition of the signaling pathways.

JNK signaling plays an important role in the regulation of apoptosis, formation, and differentiation of osteoclasts [9,63,64]. Bone marrow-derived macrophages isolated from mice lacking JNK1 or carrying a mutated form (JunAA/JunAA) of c-Jun that cannot be phosphorylated by the JNKs show reduced osteoclast differentiation and bone resorption activity [63]. Moreover, impairment of JNK signaling by overexpression of dominant-negative JNK1, c-Jun, and c-Fos, or the JNK-specific inhibitor SP600125 abrogates the anti-apoptotic effect of RANKL/RANK/TRAF6 signaling in osteoclasts [9]. This indicates that JNK/c-Jun signaling mediates the RANKL-induced anti-apoptotic process in mature osteoclasts. Blockade of JNK activity at the pre-fusion osteoclast stage results in the reversion of tartrate-resistant acid phosphatase (TRAP)-positive cells (representing pre-osteoclasts at the pre-fusion stage) to TRAP-negative cells (representing osteoclast precursors), even in the continuous presence of RANKL, demonstrating that the JNK pathway is required for maintaining osteoclastic commitment, until fusion [65]. In osteoclasts, JNK signaling pathways are reported to structurally organize as a signaling cascade (Figure 2). MAPKKKs, such as MEKK1, and transforming growth factor beta-activated kinase 1 (TAK1) stimulate the MAPKKs MKK4 and MKK7, which induce dual phosphorylation of JNK at a conserved TPY motif [9,66].

### 3.1. Upstream Activators of JNK Signaling in Osteoclasts

The osteoclastogenic factor RANKL activates JNK signaling through TRAF6, thus stimulating osteoclast differentiation [9,64]. M-CSF produces ceramide 1-phosphate, which is reported to be mitogenic for fibroblasts and acts as a lipid second messenger, in murine bone marrow-derived macrophages, and ceramide 1-phosphate from M-CSF-stimulated cells mediates their proliferation via rapid phosphorylation of protein kinase B (also known as Akt) and JNK [67,68].

JNK signaling activated by the inflammatory cytokines TNF-α and IL-1 induces cell-cell fusion to form osteoclasts and enhances osteoclast survival, respectively [69,70,71]. IL-17A facilitates autophagic activity of osteoclast precursors and promotes osteoclastogenesis via activating the RANKL-JNK pathway [72].

In JNK signaling induced by growth factors and other signals, Wnt5a, a non-canonical Wnt ligand secreted from osteoblasts, binds to its receptor, receptor tyrosine kinase-like orphan receptor (Ror2) expressed in the plasma membrane of osteoclasts. Wnt5a-Ror2 signaling induces RANK expression through JNK activation and recruitment of c-Jun to the promoter of RANK-coding gene in osteoclast precursors, thereby enhancing RANKL-mediated osteoclastogenesis [73], suggesting that Wnt5a-Ror2-JNK signaling between osteoblasts and osteoclast precursors mediates osteoclastogenesis. In addition, CCN2 (connective tissue growth factor, cystein rich protein, and nephroblastoma overexpressed gene), known as a connective tissue growth factor, directly binds to RANK or OPG to enhance osteoclastogenesis via the activation of RANKL-RANK-JNK signaling and removal of the anti-osteoclastogenic effect of OPG [74]. Lipopolysaccharide (LPS), a prominent pathogenic factor in inflammatory bone diseases, induces osteoclast formation by activating the JNK-STAT3-NFATc1 pathway via the generation of ROS as second messengers [75].

### 3.2. Upstream Inhibitors of JNK Signaling in Osteoclasts

Several inflammatory cytokines that negatively influence osteoclastogenesis via JNK inactivation have been identified. IL-3 induces the irreversible inhibition of RANK expression by downregulating JNK activation in osteoclast precursors, and thus suppresses RANKL-induced osteoclastogenesis [76,77]. IL-4 blocks RANKL-induced activation of NF-κB and JNK depending on STAT6, and IL-10 downregulates RANKL-induced expression of NFATc1, c-Jun, and c-Fos, and JNK phosphorylation, ultimately impairing osteoclastogenesis [40,78]. IL-6 suppresses osteoclast differentiation through inhibition of JNK activation, at least in part by upregulating the expression of *DUSP1* and *DUSP16*, which dephosphorylate JNK [79]. Interferon-γ inhibits RANKL-induced activation of JNK through degradation of TRAF6 in osteoclast precursors or induction of osteoclast inhibitory peptide-1 expression [80,81].

Some therapeutic agents having anti-osteoporosis, anti-tumor, and anti-inflammation activities have been reported to suppress osteoclastogenesis through JNK inactivation. The anti-osteoporotic agent estrogen and the selective estrogen-receptor modulators tamoxifen and raloxifene that mimic the anti-osteoporotic effect of estrogen suppress RANKL-induced JNK-c-Jun axis signaling, resulting in a decrease in osteoclast formation and differentiation [82,83]. The anti-tumor agent afatinib (an ATP-competitive 4-anilinoquinazoline derivative), an irreversible epidermal growth factor receptor tyrosine kinase inhibitor, has proven efficacious in phase III trials in patients with non-small cell lung cancer, which is known as the third most common cause of bone metastases [84]. This inhibitor with anti-tumor effect specifically inhibits RANKL-induced phosphorylation of JNK and Akt, ameliorating the differentiation and bone resorbing activity of osteoclasts [85]. Cepharanthine, a natural alkaloid extracted from *Stephania cepharantha* Hayata, has been used in the clinic for the treatment of tumors and inflammatory diseases [86]. This agent prevents estrogen deficiency-induced bone loss by inhibiting osteoclastogenesis via the attenuation of JNK and PI3K-Akt signaling [87]. Curcumol, a sesquiterpene and one of the major components of the essential oil of *Rhizoma curcumae* with antitumor and anti-inflammatory properties, inhibits osteoclastogenesis by specifically impairing RANKL-induced JNK-activator protein-1 (AP-1) signaling [88]. In pathological conditions, such as uremic disease, elevated serum phosphate levels are closely related with ectopic extraskeletal calcification, especially in vascular calcification [89]. A high concentration of extracellular inorganic phosphate inhibits osteoclast differentiation and bone resorption activity through specific downregulation of RANKL-induced JNK and Akt activation, with no significant changes in p38 and ERK phosphorylation [90].

### 3.3. Downstream Targets of JNK Signaling in Osteoclasts

RANKL-induced activated JNK phosphorylates the transcription factor c-Jun, which forms a complex with c-Fos, an essential transcription factor for osteoclast formation [9,91]. JNK signaling also induces the expression of the calcium/calmodulin-dependent protein kinase (CaMK), c-Fos, and NFATc1, which are involved in the maintenance of osteoclast lineage commitment [65,75]. Semaphorin 3D is a downstream target of JNK signaling and is involved in stimulating TNF-α-induced osteoclastogenesis [69].

### 3.4. Phosphatase Regulation of JNK Signaling in Osteoclasts

DUSP10, a member of the MAPK phosphatase family of dual-specificity phosphatases, predominantly dephosphorylates JNK and is induced in osteoclasts by RANKL stimulation. RANKL-induced activation of DUSP10 is thought to limit JNK signaling in order to inhibit osteoclasts from undergoing apoptosis [92]. Results obtained through in-vitro differentiation of osteoclast precursors obtained from DUSP1-deficient mice revealed that DUSP1 removes RANKL-induced phosphorylation of JNK and negatively regulates osteoclast differentiation and activation [13].

## 4. p38 Signaling in Osteoclasts

**Figure 3:**
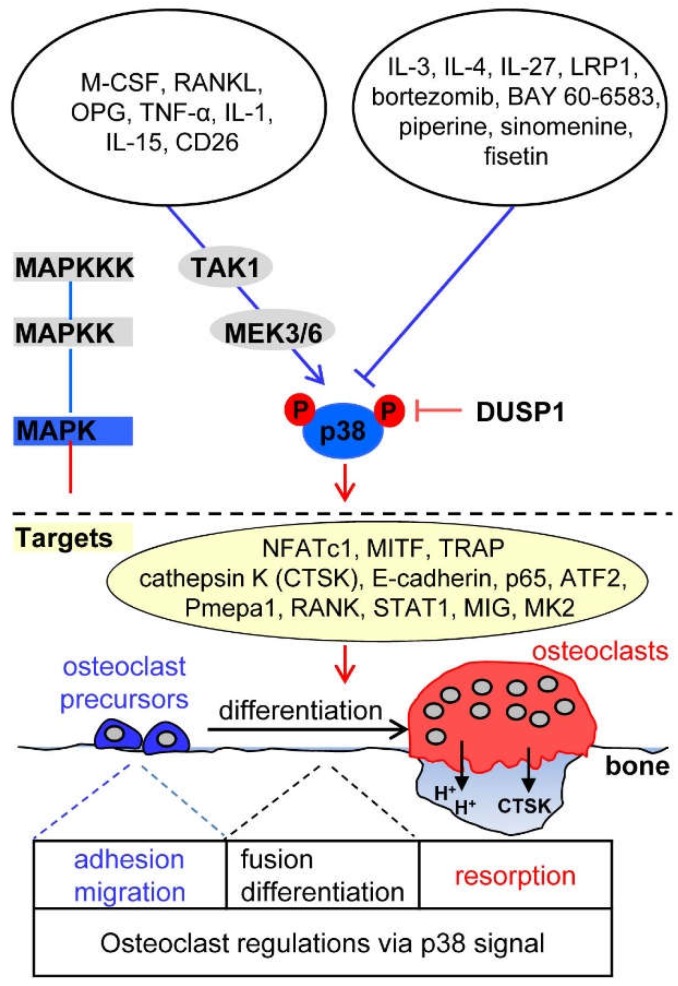
Osteoclastogenic signaling cascade controlled by upstream activators and inhibitors of p38 mitogen-activated protein kinases (MAPK). The presented molecules are described in the part of “p38 signaling in osteoclasts”. Arrows indicate activation of the signaling pathways while T bars indicate inhibition of the signaling pathways.

The p38 signaling pathway plays a key role in the regulation of osteoclast formation and maturation, and thus, in bone resorption and remodeling [5,93]. A study using conditional p38α knockout mice showed that p38α deficiency induces increased bone mass in young mice, with decreased numbers of osteoclasts and bone resorption [93]. Consistent with these in-vivo data, expression of dominant negative forms of p38α and treatment with a specific p38 inhibitor in osteoclast precursors resulted in complete blockage of RANKL-induced osteoclastogenesis in vitro [94]. Furthermore, p38α plays an important role in coupling osteoclastogenesis and osteoblastogenesis, as demonstrated by the fact that specific ablation of p38α in monocytic osteoclast precursors obtained from p38α-flox; LysM-Cre mice indirectly inhibited osteoblast proliferation and differentiation via a decrease in the expression and secretion of coupling factors, BMP-2 and platelet-derived growth factor AA, which are expressed via the p38 MAPK-Creb axis in osteoclasts [93]. In osteoclastogenesis, osteoclastogenic factors stimulate MAPKKKs, including TAK1 and relay the phosphorylation of MAPKKs, MKK3, and MKK6 (Figure 3) [95,96]. Subsequently, the activated MKK3 and MKK6 induce dual phosphorylation of p38α at a conserved TGY motif, facilitating osteoclastogenesis by the activation of NF-κB signaling and NFATc1 induction [96,97].

### 4.1. Upstream Activators of p38 Signaling in Osteoclasts

M-CSF-c-Fms signaling induces p38 activation during macrophage development [8,98,99]. The binding of RANKL to its cognate receptor RANK leads to the phosphorylation of p38 in osteoclast precursors through adaptor protein TRAF6, thus inducing osteoclast differentiation [5,94]. OPG directly activates p38 signaling, thereby potentiating osteoclast function through MMP-9 expression [24] or by retracting osteoclast adhesion structures [25].

TNF-α and IL-1 directly activate p38 in a RANKL-independent manner [100,101]. IL-15 allows a synergistic effect of RANKL-induced osteoclast formation and bone resorption activity through p38 activation [102]. The expression of CD26, a cell-surface glycoprotein with dipeptidyl peptidase IV activity, in osteoclasts is accompanied by increased activation of MKK3/6-p38-MITF signaling, which is essential for early osteoclast differentiation [103]. On the contrary of the positive role of p38 activation in osteoclastogenesis, p38 activation by any stimuli has been reported to negatively regulate osteoclastogenesis. TLR stimulation with the TLR2 agonist Pam3Cys or the TLR4 agonist LPS inhibits the differentiation of human peripheral blood mononuclear cells into osteoclasts by downregulating RANK transcription and cell-surface expression of the M-CSF receptor c-Fms. Especially, it has been shown that TLR2-induced proteolytic cleavage of c-Fms depends on both p38 and ERK activation [104]. Interestingly, serum amyloid A, which is a major acute-phase protein that is secreted from liver cells in response to infection or injury, blocks M-CSF/c-Fms signaling via activation of p38 and ERK, thus inhibiting osteoclast formation by repressing osteoclast-associated genes, such as RANK and TRAF6, and inducing expression of anti-osteoclastogenic genes, such as MafB and the gene encoding interferon regulatory factor 8 [105].

### 4.2. Upstream Inhibitors of p38 Signaling in Osteoclasts

Various reports have suggested that any stimulus can suppress RANKL-induced p38 activation and thus suppress osteoclastogenesis. IL-3 negatively regulates p38 signaling through the activation of STAT5 in the early stages of RANKL-induced osteoclast differentiation, thus inhibiting osteoclastogenesis via the upregulation of the anti-osteoclastogenic regulators Id1 and Id2 [38]. IL-4 inhibits osteoclastogenesis by specifically blocking the activation of RANKL-induced p38 and NF-κB signaling in a STAT6-dependent manner, but not M-CSF signaling [40]. IL-27 inhibits the differentiation of human peripheral blood mononuclear cells into osteoclasts by downregulating the expression of osteoclastogenic factors RANK, triggering receptor expressed on myeloid cells (TREM-2), and NFATc1, as well as RANKL-induced activation of p38, ERK, and NF-κB signaling [106].

Blockage of osteoclast formation by the TLR9 agonist CpG-ODN is attributed to reduced expression of c-Fos by shifting from RANKL-induced sustained activation of ERK, JNK, and p38 to transient activation resulting from increased expression of PP2A [53]. Ctsk-Cre;Lrp1f/f mice with osteoclast-specific deletion of low-density-lipoprotein receptor-related protein 1 (LRP1) showed dramatically decreased trabecular bone mass with significantly increased osteoclast formation. Consistent herewith, ex-vivo culture experiments revealed that LRP1-deficient bone marrow-derived macrophages from Ctsk-Cre;Lrp1f/f mice more efficiently differentiated into osteoclasts by elevating NF-κB and p38 signaling than LRP1^+/+^ macrophage cells, indicating that LRP1 negatively regulates osteoclastogenesis by blunting p38 and NF-κB signaling [107].

There exist synthetic and natural compounds that regulate osteoclast differentiation through the modulation of p38. Bortezomib, a synthetic proteasome inhibitor approved by the Food and Drug Administration for use in multiple myeloma, inhibits p38-triggered early osteoclast differentiation and thus blocks osteoclastic bone resorption [108]. Stimulation of the A2B adenosine receptor with its specific agonist BAY 60-6583 inhibits RANKL-induced NF-κB and p38 signaling and leads to a decrease in both cell-cell fusion in the late stage of osteoclast differentiation by notably reducing osteoclast fusion factors (Atp6v0d2 and DC-STAMP) and osteoclastic bone resorption [109]. Natural compounds, such as piperine and sinomenine, which are plant alkaloids, and fisetin, a flavonoid found in the smoke tree, not only show anti-angiogenic, anti-inflammatory, and anti-tumor activities, but also suppress RANKL-induced osteoclast differentiation via the downregulation of p38 activity [110,111,112].

### 4.3. Downstream Targets of p38 in Osteoclasts

Activated p38 directly phosphorylates and stimulates NFATc1 and MITF, transcription factors essential for osteoclastogenesis, inducing gene expression of osteoclastic proteins, such as TRAP, cathepsin K, and E-cadherin [113,114]. p38 activated by RANKL-TAK1-MKK6 signaling induces the phosphorylation of the NF-κB p65 subunit on Ser-536, resulting in increased transcription of NF-κB and NFATc1 [96]. In addition, RANKL/RANK/TRAF6/MKK3/6 signaling induces p38 activation followed by phosphorylation of activating transcription factor 2 (ATF2), stimulating RANKL-induced osteoclast differentiation, but not osteoclast function [115]. RANKL-stimulated active p38 strongly induces the expression of prostate transmembrane protein androgen induced 1 (Pmepa1), which subsequently upregulates cell-surface expression of RANK on osteoclasts [116]. RANKL-induced p38 activation induces STAT1 phosphorylation at Ser727 and promotes the expression and secretion of monokine induced by interferon-γ (MIG), which stimulates the adhesion and migration of osteoclast precursors and differentiated osteoclasts [117]. RANKL-stimulated active p38 directly phosphorylates MAPK-activated protein kinase-2 (MK2), which is critical for regulating the expression of osteoclastic fusion genes, DC-STAMP and osteoclast stimulatory transmembrane protein (OC-STAMP) [118].

### 4.4. Phosphatase Regulation of p38 Signaling in Osteoclasts

Mice lacking *DUSP1* exhibit drastic osteoclast activation in response to local LPS injection, and osteoclast precursors derived from *DUSP1*^−/−^ mice show increased cell-cell fusion to multinucleated osteoclasts and osteoclastic bone-resorptive activity [119]. Further, DUSP1 is expressed by RANKL stimulation, is localized into the nucleus, and preferentially dephosphorylates the threonine and tyrosine residues of activated p38 and JNK over those of ERK, thus inducing transient p38 and JNK activation in response to stress and negatively regulating osteoclast formation and function by inactivating p38 MAPK-dependent signaling [13,119].

## 5. Distinct Kinetics of MAPKs and Crosstalk between MAPKs

The strength and duration of the response of MAPKs to different exogenous stimuli determines the biological outcome of the response. For instance, epidermal growth factor-induced transient ERK activation via the Raf-MEK-ERK axis induces proliferation in PC12 neuroendocrine cells, whereas nerve growth factor-induced prolonged ERK activation via the Raf-MEK-ERK axis leads to the differentiation of PC12 cells into sympathetic neuron-like cells [120,121]. Interestingly, prolonged ERK activation by epidermal growth factor in PC12 cells overexpressing the epidermal growth factor receptor switches cell fate from proliferation to differentiation [120]. Thus, cell fate decision is regarded a consequence of the duration of ERK activation. A recent report indicated that in osteoclast precursors, MAPK signaling induced by M-CSF or RANKL differed in terms of the extent and duration of ERK, p38, and JNK phosphorylation, as well as the selective phosphorylation of JNK isoforms [8]: (i) M-CSF induced more pronounced and sustained ERK phosphorylation than RANKL, (ii) RANKL induced more and longer p38 phosphorylation than M-CSF, (iii) M-CSF favorably phosphorylated JNK1 rather than JNK2 or JNK3, whereas RANKL had no such preference, and iv) M-CSF induced immediate monophasic activation (5 to 20 min) of MAPKs, whereas RANKL induced biphasic immediate (5 to 20 min) and delayed activation (8 to 24 h) of MAPKs. The different kinetics of MAPK activation by M-CSF or RANKL are considered to be related to cell fate decision of osteoclast precursor proliferation or differentiation [8].

### 5.1. ERK Kinetics in Osteoclast Metabolism

RANKL induces sustained ERK phosphorylation and maintains increased protein levels of c-Fos, resulting in enhanced osteoclastogenesis [53]. In contrast, the TLR9 ligand CpG-ODN leads to the transition of RANKL-induced sustained ERK activation into transient ERK activation by enhancing the expression of phosphatase PP2A, thereby decreasing the c-Fos level by degrading c-Fos mRNA and protein, and consequently inhibiting osteoclastogenesis. Moreover, when osteoclast precursors were pretreated with okadaic acid, a phosphatase inhibitor, RANKL-induced transient ERK activation by CpG-ODN was reverted to persistent activation, consequently inducing c-Fos expression. Additionally, 17β-estradiol triggers osteoclast apoptosis via transient ERK activation, peaking at 5 min after estrogen administration and returning to the basal level by 30 min, but blocks osteoblast apoptosis via long-lasting ERK phosphorylation for at least 24 h [122,123]. The opposite effects of ERK activation on apoptosis were accounted as a result of the differential duration of ERK phosphorylation in the osteoclasts and osteoblasts [122,123]. Collectively, the switch between RANKL-induced transient and persistent ERK activation is able to be regulated by tuning the activity of phosphatase and TLR-mediated signaling in the osteoclast precursors or mature osteoclasts.

### 5.2. JNK Kinetics in Osteoclast Metabolism

Lissencephaly-1 (LIS1)-flox;LysM-Cre mice, in which LIS1, a key regulator of microtubules and the cytoplasmic dynein motor complex, is specifically deleted in myeloid cells relevant to osteoclast precursors, exhibit increased bone mass, due to defective osteoclast formation and bone resorption [124]. Consistent with these findings in vivo, osteoclast precursors derived from LIS1 conditional knockout mice exhibited impaired osteoclast formation and accelerated apoptotic cell death through the suppression of M-CSF-induced prolonged ERK activation and the induction of RANKL-induced prolonged JNK activation. Moreover, the ablation of RelA, a component of NF-κB, induced strong activation of JNK by RANKL and resulted in JNK-Bid-mediated apoptosis of osteoclast precursors [92]. Together, these results indicate that changes in the activities of ERK and JNK during M-CSF- and RANKL-mediated osteoclastogenic signaling regulate the apoptosis of osteoclast precursors.

### 5.3. p38 Kinetics in Osteoclast Metabolism

p38α-flox;LysM-Cre mice exhibit bone defects in an age-dependent manner, displaying osteopetrosis at 2.5 months and osteoporosis at 6 months of age [93]. When compared with the differentiation of osteoclast precursors obtained from 2.5-month-old wild-type mice, osteoclast precursors isolated from age-matched p38α-deficient mice showed increased osteoclast formation at low cell density, but decreased osteoclast formation at high cell density. Hotokezaka et al. suggested that ERK inactivation induces RANKL-induced strong p38 activation and positively regulates osteoclastogenesis via the inhibition of ERK-mediated osteoclast precursor proliferation [125]. We also reported that p38 activation via the RANKL-RANK-TRAF6 axis leads to a shift from proliferation to differentiation in osteoclast precursors [8]. Therefore, the positive role of p38 in RANKL-, but not M-CSF-induced osteoclastogenesis seems to be differential in osteoclast formation and bone remodeling, according to spatial conditions of cell-cell confluency and physiological development stage, respectively.

### 5.4. Crosstalk between ERK and p38 in Osteoclast Metabolism

ERK inactivation by PD98059, a specific MEK inhibitor, suppressed serum-stimulated proliferation of SaOS-2 human osteosarcoma cells, but stimulated the osteogenic differentiation of these cells via accelerated p38 activation [126]. This phenomenon could be explained by a competition and balancing of p38-induced cell differentiation and MEK/ERK-mediated cell proliferation. In accordance herewith, ERK inactivation in osteoclast precursors by treatment with MEK inhibitors (U0126 and PD98059) elevated RANKL-induced p38 activation and resulted in enhanced osteoclast [125]. In addition, treatment of osteoclast precursors with p38 inhibitors (SB203580 and PD169316) induced an increase in RANKL-induced ERK activation and led to decreased osteoclast differentiation. These results suggest that MEK/ERK and p38 pathway may be involved in the suppression and induction of osteoclastogenesis, respectively, by regulating a seesaw-like crosstalk between ERK and p38 MAPK signaling. Of note, PD98059 and U0126 were found to show off-target effects on the MEK5/ERK5 pathway at higher concentrations, suggesting the possibility that ERK5 may contribute to some of the roles ascribed to ERK1/2 in osteoclastogenesis [127].

## 6. Conclusions

**Figure 4:**
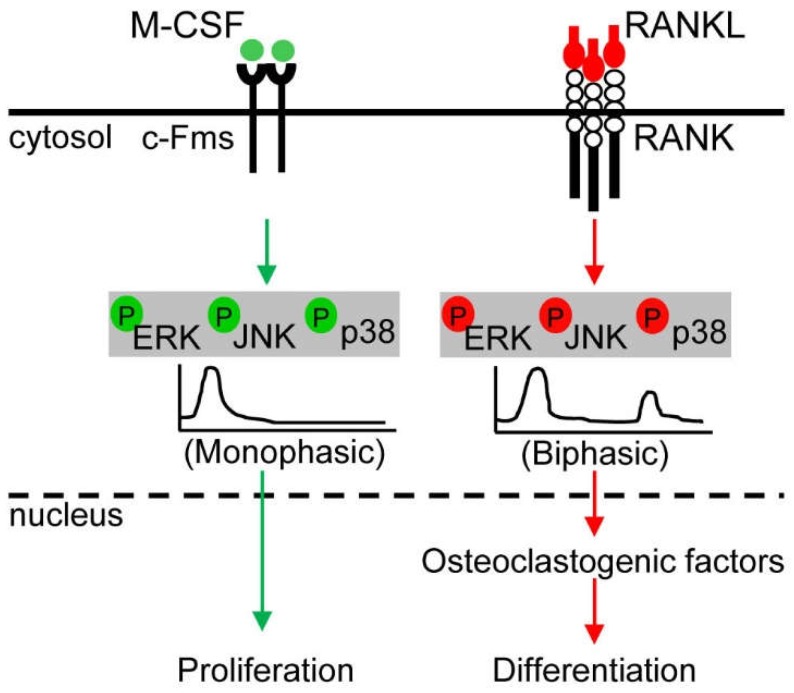
Osteoclast precursor proliferation by macrophage colony stimulating factor (M-CSF)/c-Fms-mediated monophasic activation of MAPKs and osteoclast differentiation by receptor activator for nuclear factor κ-B ligand (RANKL)/RANK-mediated biphasic activation of MAPKs. Arrows indicate activation of the signaling pathways, a solid line indicates the plasma membrane, and a dotted line indicates the nuclear membrane of osteoclast precursors. Green color means the signaling pathway induced by M-CSF and red color means the signaling pathway induced by RANKL.

M-CSF and RANKL act as osteoclastogenic key regulators in normal osteoclast metabolism and share ERK, JNK, and p38 as signal mediators, but exhibit differences in the extent and duration of activation and MAPK isoform specificity. Moreover, M-CSF induces monophasic activation with an immediate phosphorylation (5 to 20 min) of MAPKs; distinctively, RANKL leads to biphasic activation with both immediate (5 to 20 min) and delayed phosphorylation (8 to 24 h) of MAPKs. The timing of RANKL-induced delayed MAPK activation coincided with the onset of osteoclast differentiation. Thus, the differential MAPK signaling induced by M-CSF and RANKL is recognized to determine the osteoclast precursor proliferation and osteoclast differentiation, respectively (Figure 4) [8]. JNK and p38 activated via RANKL-RANK signaling predominantly mediate osteoclastic apoptosis and promote osteoclast differentiation and function, respectively, whereas ERK activation via M-CSF/c-Fms axis preferentially potentiates osteoclast precursor proliferation [4,6,7,8,9,128,129]. Because p38 signaling is more tightly connected to the control of osteoclast metabolism than ERK and JNK signaling, researchers have tried to apply p38 inhibitors to prevent periopathogen-induced periodontal and active alveolar bone loss with degradation of mineralized and non-mineralized tooth tissues [130,131,132] and to treat rheumatoid arthritis with synovial inflammation, overactive osteoclast function, cartilage degradation, and bone erosion [133]. Although p38 is currently considered as a potential therapeutic target for inflammation-mediated bone loss [134], osteoclast-specific indirect regulators of p38 rather than direct p38 inhibitors should be developed to avoid side effects to other tissues and cells. Further studies are needed to clarify the detailed molecular mechanism underlying the crosstalk between MAPKs and the regulation of MAPKs by the balancing of kinases and phosphatases, and to explore the roles of specific isoforms of JNK1/2/3 and co-modulators capable of tuning p38 MAPK cascades in osteoclast metabolism.
